# Temporal Patterns of Phenotypic Antimicrobial Resistance and Coinfecting Pathogens in *Glaesserella parasuis* Strains Isolated from Diseased Swine in Germany from 2006 to 2021

**DOI:** 10.3390/pathogens11070721

**Published:** 2022-06-24

**Authors:** Isabeau Wiencek, Maria Hartmann, Jörg Merkel, Sara Trittmacher, Lothar Kreienbrock, Isabel Hennig-Pauka

**Affiliations:** 1 Field Station for Epidemiology, University of Veterinary Medicine Hannover, 49456 Bakum, Germany; isabeau.anna-katharina.wiencek@tiho-hannover.de (I.W.); sara.trittmacher@tiho-hannover.de (S.T.); 2 Department of Biometry, Epidemiology and Information Processing, WHO Collaborating Centre for Research and Training for Health at the Human-Animal-Environment Interface, University of Veterinary Medicine Hannover, 30559 Hannover, Germany; maria.hartmann@tiho-hannover.de (M.H.); lothar.kreienbrock@tiho-hannover.de (L.K.); 3 Department of Infectious Diseases, Institute for Microbiology, University of Veterinary Medicine Hannover, 30173 Hannover, Germany; joerg.merkel@tiho-hannover.de

**Keywords:** Gps serotypes, antibiotic resistance, endemic infection, Glaesser’s disease, respiratory disease, influenza virus, porcine reproductive and respiratory syndrome virus, *Streptococcus suis*, susceptibility

## Abstract

*Glaesserella parasuis* (Gps) causes high economic losses in pig farms worldwide. So far no vaccine provides cross-protection for different serotypes, so antibiotic treatment is widely used to cope with this pathogen. In this study, routine diagnostic data from 2046 pigs with Gps related diseases sent for necropsy to a German laboratory in the time period 2006–2021 were analysed retrospectively. In the time period 2018–2021, the most frequent serotypes (ST) detected were ST4 (30%) and ST13 (22%). A comparison of the reference period 2006–2013 prior to obligatory routine recording of antimicrobial usage in livestock with the period 2014–2021 resulted in a statistically significant decrease of frequencies of resistant Gps isolates for ceftiofur, enrofloxacin, erythromycin, spectinomycin, tiamulin and tilmicosin. While in 2006–2013 all isolates were resistant for tetracyclin and cephalothin, frequencies of resistant isolates decreased in the second time period to 28% and 62%, respectively. Parallel to the reduction of antimicrobial usage, during recent years a reduction in resistant Gps isolates has been observed, so only a low risk of treatment failure exists. Most frequently, pigs positive for Gps were also positive for *S.suis* (25.4%), PRRSV-EU (25.1%) and influenza virus (23%). The viral pathogens may act as potential trigger factors.

## 1. Introduction

Glässer’s disease caused by *Glaesserella parasuis* (Gps) has been known for more than 120 years as a primary porcine pathogen [1]. Fifteen serotypes (ST) can be differentiated so far based on variations in their capsule loci [2,3]. The systemic disease is dominated by polyserositis, meningitis and arthritis and often initiated by external trigger factors, as, e.g., transportation or viral infections. On the other hand, Gps belongs to the physiological normal commensal bacteria of the upper respiratory tract, so all pigs can be considered as positive for this bacterium [4]. While piglets are colonized during their first days of life by contact with the sow, nursery pigs are most often affected by disease because several factors serve as potential trigger factors in this age group paralleled by a decline in passive maternal immunity [5]. Research on virulence characteristics and factors to differentiate between commensal and virulent strains have led to the development of new diagnostic procedures in addition to serotyping. A PCR diagnostic based on the virulence-associated trimeric autotransporters (vtaA) or the leader sequence as a specific domain of this gene were used to predict virulence of Gps strains. A Pathotyping-PCR based on 10 putative virulence marker genes was developed from genome-wide virulence association studies [2]. Recently, a comparison of these methods and the distribution of genetic virulence markers in the different serotypes was published [6]. Virulence markers were more frequently found in strains isolated from systemic sites of infection, but not fully consistent with functional assay results as, e.g., phagocytosis and serum susceptibility tests [7]. Gps causes pneumonia under circumstances not specified so far, so that especially for samples from the respiratory tract a virulence marker would be supportive for the diagnostic procedure [8,9]. In total, 91% of the isolates and especially most isolates from the respiratory tract were classified as virulent using the methods mentioned above [6]. 

It is hypothesized that the composition of mucosal surface microbiota of the upper respiratory tract is decisive for maintenance of health in pigs endemically infected with Gps. Pigs from healthy farms showed higher richness and diversity in bacterial species of the nasal microbiota compared with pigs from farms with disease caused by Gps [10]. The microbiome of the different effector sites as upper and lower respiratory tract, and also the intestine, is highly decisive for maintenance of homeostasis and immunity [11]. Microbial communities in the upper respiratory tract are considered to reduce the colonisation with pathogenic microorganisms by competitive exclusion [12,13]. On the other hand, the microbiome of tonsil and nasal cavity in pigs was found to bear a risk for respiratory disease [10,14,15]. Less-adapted, pathogenic microorganisms can be a source of infection after translocation from the upper to the lower respiratory tract, which is also evident for Gps. In the lungs of healthy individuals translocated pathogens are considered not to proliferate after microaspiration (neutral dispersal model) because they are cleared before becoming resident [13,16]. However, any shifts in the microbiome by, e.g., acute virus infection or long-lasting immune dysfunction might disrupt this balance in airway clearance [13]. Antibiotic treatment can also disturb this balance [10]. After invasion, Gps can be spread from the lungs to systemic sites [17]. In many asymptomatic pig populations, Gps colonizes the mucosal surfaces and is suggested to be embedded in its biofilm. This biofilm might protect Gps from antimicrobial treatment, so it cannot be eliminated successfully in carrier pigs [18]. Nevertheless, under field conditions, the most important measure to control disease is antibiotic treatment of individuals or metaphylactic treatment of groups. The widely used metaphylactic treatment of suckling piglets early in life or at weaning to prevent disease is problematic. Due to the fact that the development of active immunity depends on an intact microbiome and exposure to colonizing Gps, antibiotic treatment can result in losses later in life [19]. 

Retrospective data of Gps isolated from diseased swine in north-western regions in Germany sent for necropsy within the last 16 years were evaluated with regard to antimicrobial resistance and coinfections. Passive surveillance report on antimicrobial usage (AMU) and resistance (AMR) data in the time period 2008–2015 preceded the obligatory monitoring of AMU starting in 2014 as fixed in the 16th Amendment of the German Pharmaceuticals Act. Already since 2011 the treatment frequency with antimicrobial substances was reduced in German swine farms [20,21,22]. AMU differs between age groups and indication. In nursery pigs, the age groups most often affected by Gps respiratory diseases were most often treated with tetracyclines and amoxicillins [23]. It can be hypothesized that the general reduction of AMU in swine might have led to a decrease of resistant Gps isolates and finally outweighed the common targeted metaphylactic usage of antimicrobials to prevent disease caused by Gps. To follow this hypothesis, frequencies of Gps isolates resistant against most important antimicrobial substances originating from the time period 2006–2013 prior to monitoring of AMU were compared with those from the time period 2014–2021. Study results should allow assessment of the risk of treatment failure in the future and indicate the still most effective therapeutics to be used in case of disease. In addition, most frequent combinations with other microorganisms were identified as potential important biotic trigger factors, which should also be addressed by preventive measures on farms.

## 2. Results

### 2.1. Meta Data of Sample Set and Microbiological Findings for G. parasuis

In total, data from 2046 pigs out of 1581 farms were evaluated retrospectively for the time period 2006–2021. All these pigs were selected for evaluation because they had been found to be positive for Gps by bacteriological culture. Most isolates (*n* = 1760, 86%) originated from the surrounding area of the diagnostic laboratory. The age group of sampled pigs was recorded for 1740 (85%) of the samples resulting in 86.0% of isolates originating from growing pigs (nursery), 9.2% (*n* = 188) of isolates from suckling piglets and 4.5% (*n* = 92) and 0.3% (*n* = 6) originating from fattening pigs and gilts, respectively. Most strains (*n* = 1698, 83%) were cultured from the respiratory tract. From other organs, Gps was isolated with descending frequencies as follows: 6.8% (*n* = 139) from pericardium, 4.1% (*n* = 84) from the pleural cavity, 3.4% (*n* = 70) from the brain, 1.4% (*n* = 29) from joints, 1.1% (*n* = 23) from the abdominal cavity and 0.2% (*n* = 4) from the nose. 

In the years 2018–2021, typing of Gps isolates was performed with multiplex PCR to determine the respective serotype. In a subgroup of 101 isolates (4.9% of the whole data set), the serotype was defined resulting most often in serotype 4 (30%) followed by serotypes 13 (22%), 7 and 5/12 (13%), as shown in Table 1. In the respiratory tract, all Gps serotypes were found. From other organ sites, only serotypes 4, 7, 9, 5/12 and 13 were isolated. From brain, only serotypes 5/12 and 13 were isolated. More than 50% of the seven Gps serotype 9 were isolated from heart, pleural and abdominal cavity. The distributions of the Gps isolates with regard to age group and organ site are shown in Table 2. A subset of 54 Gps strains was selected randomly from different organs, covering different serotypes and tested by PCR for the virulence associated marker gene *vta.* Approximately 50% of the strains were from nursery pigs. All strains were positive for *vta* (100%). The distributions of the positive strains are shown in Table 3.

### 2.2. Distribution of Antimicrobial Resistance 

The distribution of Minimum Inhibitory Concentrations (MIC) of selected antimicrobials and the respective number of Gps isolates is shown in Table 4. Resistant isolates were found for all tested substances, with lowest frequencies of resistant isolates for ceftiofur (*n* = 36, 1.8%), colistin (*n* = 72, 3.5%), enrofloxacin (*n* = 23, 1.1%), florfenicol (*n* = 4, 0.2%), spectinomycin (*n* = 51, 2.5%), tiamulin (*n* = 55, 2.7%), tilmicosin (*n* = 84, 4.1%) and tulathromycin (*n* = 2, 0.6%). Frequencies of resistant isolates for most commonly used antimicrobials, penicillin, ampicillin and tetracyclin, were 13.6%, 11.2% and 16.8% (*n* = 278, *n* = 229, *n* = 344), respectively. The highest frequency of resistant isolates was found for erythromycin (*n*= 886, 43.3%).

All 70 isolates originating from the brain and all 28 isolates originating from joints were susceptible for ceftiofur, florfenicol and tiamulin. The nine isolates from the brain and the seven from joints, which were tested for tulathromycin, were also fully susceptible. In addition, all isolates from joints were fully susceptible for tilmicosin. For most tested substances, the interquartile ranges of MICs were narrow with an exception for erythromycin (0.12–1.0 mg/L), penicillin (0.06–0.25 mg/L) and tetracyclin (0.5–2 mg/L).

### 2.3. Temporal Trends in AMR

**Figure 1:**
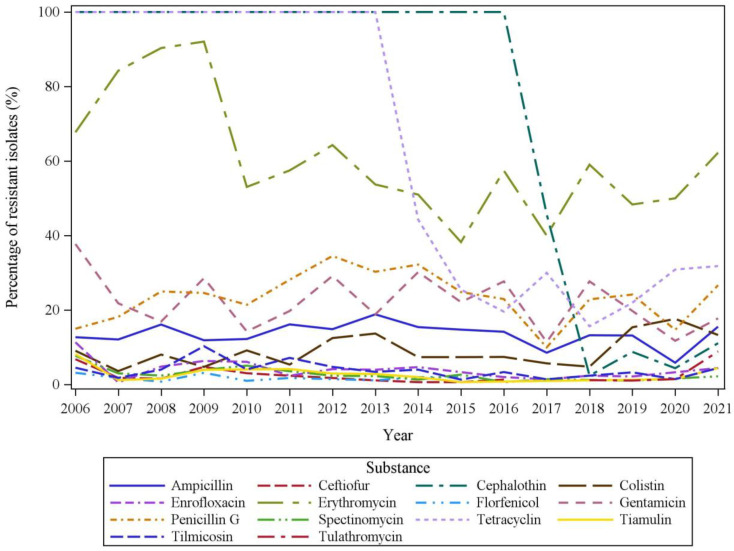
Frequencies of resistant isolates in the years 2006–2021 (*n* = 2046).

The numbers of resistant Gps isolates were compared for the different substances between the years in the time period 2006 to 2021 (see Figure 1). All isolates classified as intermediate or resistant to a respective antimicrobial according to the clinical cut-off were considered as resistant for statistical evaluations. 

The course of proportions of resistant Gps isolates throughout the years 2006–2021 reflects decreasing tendencies for most substances with some minor inconsistencies (Figure 1). 

The frequencies of resistant Gps isolates were compared between the two eight-year time periods 2006–2013 as the reference period and 2014–2021 in uni- and multivariable logistic regression models. Results for considering these time periods as a fixed factor are summarized in Table 5. For ceftiofur, enrofloxacin, erythromycin, spectinomycin, tiamulin and tilmicosin, a statistically significant decrease of resistant isolates was found in the time period 2014–2021 compared to 2006–2013. The model was not appropriate for cephalothin and tetracyclin because for both substances all isolates were resistant in the time period 2006–2013. An increase of resistant isolates was observed for no substances.

### 2.4. Comparison of AMR in Gps Isolates from Different Age Groups and Sampling Sites

Due to the fact that for some substances the comparison of frequencies of resistant isolates resulted in significant differences between age groups and sampling sites, a multivariable logistic regression model was performed. Results are shown in Table 6. For colistin, a significantly higher frequency of resistant isolates was found in the pericardium (15.8%) compared to the respiratory tract (7.7%). In contrast to that for erythromycin, a lower frequency of resistant isolates was found in isolates from the pericardium (54%) and from the joints (43%) compared to those from the respiratory tract (63%). A similar difference was found for tetracyclin with 58% (pericardium) and 59% (brain) resistant isolates compared to 74% from the respiratory tract (Table 6). 

The largest subgroup of isolates originated from the respiratory tract from nursery pigs (*n* = 1252). Comparing the time periods 2006–2013 and 2014–2021, significant decreases in resistant isolates were found for ceftiofur, erythromycin, spectinomycin and tiamulin in this subgroup (Table 7). While in the period 2006–2013 all Gps isolates were resistant to cephalothin and tetracyclin, this proportion was reduced to 59% and 27% retrospectively.

### 2.5. Parallel Detection of Other Microorganisms

After necropsy, further diagnostic procedures were performed depending on the macroscopic findings and the anamnestic report. All pigs for which isolation of Gps was successful were also tested for other bacteria in the same anatomical site Gps was isolated from. Viral infections with the Porcine Circovirus 2 (PCV2) and the Porcine Reproductive and Respiratory Syndrome Virus (PRRSV) were recorded independently of the sampling site because these pathogens cause a viraemia. Influenza virus infection, although locally in the respiratory tract, was always recorded. The frequencies of pathogens, which were detected in parallel to Gps, are shown in Table 8. The most frequent microorganisms detected in parallel to Gps were *Mycoplasma* (*M.*) *hyorhinis* (45%) and *Streptococcus* (*S*.) *suis* (25%) within the examined cases. In 23% of the pigs, an influenza virus infection was also recorded and in 25% a PRRSV infection.

In 797 cases, no other agent than Gps was found in the respective samples. In total, up to seven other pathogens were detected in parallel. From the 190 detected combinations of pathogens, the combination only with *S.suis* (10.8%), followed by an infection only with influenza virus (4.2%) were most frequent. The next most frequent combinations were only with *Bordetella bronchiseptica* (4.1%) and with PRRSV (3.8%). Less frequent combinations were with two or more agents. In Gps and *S.suis* combinations, often several other pathogens were involved. Most frequently, *Pasteurella multocida* was found as a third pathogen (8.9% of pigs with Gps and *S.suis* combination). Third pathogens in pigs infected with Gps and influenza were most frequently *M. hyorhinis* (11.9%) or *S.suis* (6.4%). Additional pathogens found in pigs infected with Gps and PRRSV were also *S.suis* (11.3%) and *M. hyorhinis* (6.6%).

In 35.5% of samples from the respiratory tract, only Gps alone was detected. In 10% of the samples, *S.suis* was also found and 4.2% of the samples were also positive for influenza virus. In total, 185 different pathogen combinations with maximum seven agents were found in the respiratory tract. In the pericardium, a monoinfection with Gps was found in 42.5% of the samples, followed by a dual infection with *S.suis* in 21.6% of the samples. A similar result was found for the pleural and the abdominal cavities with Gps monoinfection in 54% and 78% of the samples, respectively, and a dual infection with *S.suis* in 10.7% and 8.7% of the samples, respectively.

A total of 73% of the brain samples and 71% of the joint samples were only positive for Gps, followed by a dual infection with *S.suis* (brain 11.4% and joint 7%).

## 3. Discussion

In this study, routine samples positive for Gps from pigs with different clinical signs were evaluated with respect to antimicrobial resistance and parallel detection of other pathogens. The samples were taken in the years 2006–2021. The retrospective analyses of data revealed that most isolates (*n* = 1252) were harvested from the respiratory tract of nursery pigs. In total, 83% of all Gps strains were isolated from the respiratory tract. This high proportion of respiratory tract isolates is a drawback of the study because most of them might be commensal strains translocated from the upper respiratory tract. Nevertheless, to know the antimicrobial susceptibility pattern in these respiratory isolates is of high value. The largest sample set with 1252 Gps isolates originated from nursery pigs with respiratory diseases. This age group is most often treated with antimicrobial substances against respiratory but also gastrointestinal diseases [23]. Next to the target pathogen, which might be mostly *S.suis* or *E.coli*, commensal Gps strains are also affected by antimicrobial treatment, leading to an increased risk of development of antimicrobial resistance. Monitoring of antimicrobial resistance in livestock is focused on commensal bacteria because they are considered to be the reservoir for antimicrobial resistance genes that can be transferred to the human population. A higher proportion of Gps isolates from the respiratory tract than from some systemic sites was resistant against tetracycline, which is one of the most frequently used antimicrobials in swine. The decrease of proportions of resistant Gps isolates over time against most antimicrobials in general, but especially against tetracycline, is of high impact for livestock medicine. This finding supports the German strategy of reduction of antibiotic usage in livestock animals. Considering the Gps isolates from the respiratory tract as commensal organisms, they were important for the evaluation of antimicrobial resistance in this study, but less for the evaluation of coinfections. In 16.8% of the cases, a systemic infection with Gps by isolation from inner organ sites as joints and brain could be diagnosed. This resulted in 344 Gps isolates from systemic sites, which is larger than that of other studies [6]. It has been described that prior to systemic infection, Gps is located in the respiratory tract. Especially virulent Gps strains are considered to enter the blood stream after disintegration of the mucosal barrier [24,25]. The major drawback of this study evaluating the parallel detection of pathogens in the respiratory tract is the fact that no differentiation between potential virulent Gps strains involved in disease pathogenesis and harmless colonizers translocated from the upper to the lower respiratory tract is possible. Sialylated lipooligosaccharides in the outer membrane of the bacterium interact with Siglec1 on pulmonary alveolar macrophages (PAMs) followed by increased expression of TGFβ1. TGFβ1 decreased the epithelial tight junctions, which facilitates invasion throughout the respiratory epithelial barrier [26]. Virulent Gps are characterized by resistance to phagocytosis by PAMS due to production of capsule and virulence-associated trimetric autotransporters (VtaA) as surface-exposing proteins [27,28]. VtaA expression was shown in vivo and correlated to the invasiveness of Gps strains [29,30]. VtaA is therefore considered as a diagnostic marker for differentiation of commensal Gps from those with pathogenic potential, which is relevant for a comprehensive control strategy [6]. Especially for the characterization of Gps isolates from the respiratory tract, this diagnostic marker was assessed to be of practical value to collect the correct isolates for resistance testing and the production of autogenous vaccines. In our study, a subset of 54 strains was examined for the *vta* gene with 100% positive results, which indicated that all Gps strains irrespective of the organ from which they were isolated had the potential for causing disease. This is in contrast to the study of [6], in which so called “carrier isolates” from nasal swabs from pigs with no history of Gps were included in the examination for prevalence of the *vta* gene. In that study, serotypes 6 and 9 isolates were found to be often carrier isolates. In our study, carrier isolates were missing because Gps isolates originated only from diseased animals. About 13% serotype 5/12 were within the strain collection from 2018–2021. As in the study of [6], serotype 4 and 13 were found most frequently. Serotype 4 is also the most prevalent serotype in China in pigs suffering from Glässer’s disease [31], while in nasal swabs of healthy pigs most frequently serotype 7 (20%) and 3 (15%) were detected [32]. In a recent study concerning the distribution of Gps serotypes in different regions of the world, serotype 5/12 was the most common sort in Canada, China and Vietnam (22–31%) and the second most common sort in Europe (14%) behind serotype 4 (17%) [33]. These findings were comparable to those in our study, although a high proportion of Gps had been isolated from the respiratory tract. In most studies, Gps serotype 5 was found more frequently in systemic internal sites [33]. In our study, more than 90% of the Gps serotype 5 strains were isolated from the respiratory tract. 

Regarding the high number of other microorganisms in parallel to Gps in the respiratory tract in our study, a further classification of Gps strains from this anatomical site according to virulence could be useful to prioritize the found pathogens with regard to measures to be taken. In the case of infection with the viral pathogens influenza virus A and PRRSV, which occur in approximately one quarter of the animals in our study, it makes sense to address first the primary viral pathogens by implementation of preventive measures.

The basic hypothesis of the study was that a general reduction in AMU in Germany would be reflected by a decrease in the frequency of resistant isolates over time. 

In this data set a statistically significant decrease and no increase in resistant Gps isolates was found in the time period 2014–2021. In the German VetCab project in 2014, a significant reduction in treatment frequencies was already recorded in swine paralleled by strengthening the German Pharmaceutical Act [20,23,34]. Not only antimicrobial treatments but also several other factors such as, e.g., cleaning and disinfection protocols, swine density, and flooring influence the frequency of resistant bacterial isolates [35]. In this study, next to time period for some substances, other factors were also found to affect the frequency of resistant isolates. Colistin-resistant isolates were found more frequently in the pericardium than in the respiratory tract. Colistin is never used for treatment of Gps but only for gastrointestinal diseases. Effective colistin concentrations might occur in the saliva and come into contact with tonsillar Gps. Hypothetically, virulent strains could become resistant and translocate to the pericardium. An opposite effect was found for erythromycin with less resistant isolates in joints, and for tetracyclin with less resistant isolates in the pericardium and the brain compared to the respiratory tract. For florfenicol, no resistant isolates were found in inner organs with the exemption of the pleural cavity. An age effect was found for florfenicol with a higher frequency of resistant isolates in fattening pigs. Florfenicol is commonly used for intramuscular treatment of fattening pigs in the case of respiratory disease because it is highly effective against most important bacterial respiratory pathogens (*Pasteurellaceae*) and has an acceptable withdrawal time after treatment, which is especially important in slaughter-age pigs [36]. In this age group, systemic Gps disease is very rare, which is also reflected by there being the highest percentage of Gps isolates in fatteners from the respiratory tract (77%). The main indications for antibiotic usage in swine are respiratory diseases, which in nursery and fattening pigs are mainly treated with tetracycline and amoxycillin [23]. Although the most important control measures against Gps-related disease is antibiotic treatment, frequencies of resistant isolates decreased by time in this evaluation. This is in contrast to other regions, where multidrug-resistant Gps are relevant in swine production [31]. In Gps from nasal swabs of healthy pigs in China, elevated MICs with bimodal distribution were found for several antibiotic substances, suggesting the presence of non-wildtype strains [4]. For the third generation macrolide antibiotic tilmicosin, an MIC 90 of 64µg/mL was found in that study, which is eight-fold higher than that defined in our data set. A comparison of frequencies of resistant isolates between different studies is difficult because the methods for sampling, MIC determination and the chosen clinical cut-offs vary. In the study [37], chosen clinical cut-offs for interpretation of MICs from Gps isolates from Spain and the United Kingdom (UK) were different from those chosen in our study. Nevertheless, most of the MIC_50_ and MIC_90_ values were higher in the isolates from Spain and the UK than in our study. In contrast to that, MIC distribution frequencies for Gps from pigs across Europe in 2009–2012 revealed much lower MIC_50_ and MIC_90_ values for all substances tested than in our study [38].

Generally, antibiotic therapy can negatively affect physiological and competitive bacterial flora of the upper respiratory tract (e.g., nasal microbiota), impacting colonization with Gps, which is necessary for the development of a protective immunity against virulent Gps strains [19]. For the decision to treat especially young piglets with antibiotic substances, the negative effects on the development of the immune system should be taken into account. Due to the European strategy to minimize antibiotic treatment in animals and humans and strengthening of the Pharmaceutical Act, veterinarians are becoming more aware of the disadvantages of metaphylactic treatment. Among the practitioners, a high commitment to an antibiotic reduction strategy can be observed, which has led to a reduction in antibiotic usage of more than 60% since 2011, also in the livestock-dense regions in the north-western part of Germany [39]. Nowadays, practitioners focus more on other strategies, such as, e.g., use of commercial or autologous vaccines, avoidance of management and husbandry stressors, prevention of coinfections and more strict separation between different age groups [5,40]. 

An inactivated vaccine based on serotype 5 is currently available in Germany, but other serotypes cause also disease, so autogenous vaccines are commonly used. The protective effect of autogenous vaccines highly depends on the selection of the correct strain responsible for disease outbreaks on the farm to be included in the vaccine [41]. Furthermore, different Gps strains differ in their immunogenicity [42]. In general, a drawback of autogenous vaccines is the required multiple immunizations to generate long-term protection [43]. While only inactivated vaccines have been used in the field so far, some subunit vaccine candidates have been tested under experimental conditions [41]. In a recent study, the effect of a sow vaccination with a Gps antigen on the composition of the nasal microbiota of piglets was reported [44]. This finding might be due to lower pathogen shedding by the sow and increased transfer of specific maternal antibodies by colostrum as a consequence of vaccination. The VtaA proteins are considered as promising vaccine candidates for future research [45]. A controlled exposure of pigs to living Gps resulted in a higher protection than vaccination, which supports the importance of interaction of the immune system with colonizing strains for protection [46].

Due to the fact that up to now no protective vaccines are available, practitioners’ prevention strategies are aimed primarily at measures against co-infecting pathogens as important trigger factors for disease. In a recent meta-analysis of published data, it was shown that the effects of coinfections with Gps differ between in vitro and in vivo experiments [47]. Gps had no effect on PRRSV replication in vitro, but led to an increase of influenza virus and PCV2 replication in vivo. In vitro PRRSV had a decreasing effect and influenza virus had no effect on Gps replication, but an increase in Gps replication was found for both viruses in vivo. A coinfection with *Bordetella bronchiseptica* led to an increase in Gps replication. More severe clinical signs were observed in Gps-infected pigs coinfected with PRRSV, PCV2 or *Bordetella bronchiseptica* [47]. In our study, the highest proportions of coinfections were found for PRRSV, for influenza virus and for the combination with *S.suis* in approximately 25% of pigs. To our knowledge, so far no coinfection experiments of Gps and *S.suis* have been performed in pigs, although both pathogens usually cause disease in the same age group. In combined experimental infections of tracheal epithelial cells and primary alveolar macrophages with *S.suis* serotype 2 and different Gps strains, no effects on adhesion or cell invasion were found compared with the respective monoinfections [48]. The authors suggested different cell receptors involved in adhesion and invasion for both pathogens. An in vitro infection with Gps and a highly pathogenic (HP-) PRRSV strain led to induction of a severe inflammatory response in PAMS [49] and an increased proliferation rate of Gps in blood and tissue [49]. The immunosuppressive effect of a decrease of lymphocytes after a PCV2 infection led to more severe symptoms when pigs were coinfected with a weakly virulent Gps strain. In addition, Gps surface components stimulated cells of the monocyte/macrophage lineage, which triggered PCV2 replication [50]. 

The major drawback of the study is the high proportion of isolates from the respiratory tract, which cannot be differentiated between harmless colonizers and strains involved in disease pathogenesis. Only in the respiratory tract was parallel detection of other pathogens evaluated in this study because in systemic sites with some exemptions only single infections with Gps were found. For this reason, the role of other pathogens found in the respiratory tract cannot be assessed in the end. The primary viral pathogens might impact disease pathogenesis and should be addressed by preventive measures to avoid clinical outbreaks with Gps. In addition, the study suggests that the general reduction of antibiotic usage in the German livestock population is paralleled by a decrease in resistant Gps isolates. 

## 4. Materials and Methods

### 4.1. Sample Collection and Isolation of Bacterial Strains

During routine diagnostics at the Field Station for Epidemiology of the University of Veterinary Medicine Hannover in Bakum, bacteriological examinations were carried out in pigs with gross lesions such as pleuritis, pericarditis, peritonitis, serositis, arthritis, meningitis, pneumonia or suspicion of systemic disease or Glässer’s disease. 

Results from samples of diseased pigs were retrospectively evaluated in the period from 1 January 2006 to 31 Deccember 2021 in the cases in which Gps could be isolated. Available data on age group, organ site of Gps isolation and coinfecting agents were allocated to the results of antimicrobial resistance testing of the respective isolate. To guarantee independent Gps strain information for statistical evaluations within one calendar year, only the first Gps isolate from a respective farm was included in the final data set. This procedure resulted in Gps strains from 1581 farms. From 1220 farms, only one Gps strain per farm was included in the data set. A total of 826 Gps strains originated from 361 farms which had been sampled more than once within the time period 2006–2021, but with approximately a 1-year distance in time between samplings. An overview of Gps strains with available additional information is given in Table 2.

In respiratory tract infections, samples for bacteriological examination were routinely taken from altered lung tissue, including a lobar bronchus as well as the lung periphery. Other organ sites were swabbed on their surfaces (pericardium, pleural and abdominal cavity, brain, synovia (joints)) if there was suspicion of inflammation. Swabs for bacteriological culture were immediately processed in the laboratory. Swabs for PCR testing for respective pathogens were either processed right away or frozen at −20 °C until testing.

Bacteriological culture of sample smears was performed on CNA blood agar (Becton, Dickinson and Company, Sparks, NV, USA), chocolate blood agar (NAD, Blood Agar No. 2, Becton, Dickinson and Company, Sparks, NV, USA), Gassner agar (OXOID, Hampshire, United Kingdom) and Columbia agar containing 5% sheep blood (Becton, Dickinson and Company, Sparks, NV, USA). Incubation was carried out at 37 °C under standard atmosphere for 48 h, whereas the incubation of the chocolate blood agar was carried out under 8% CO_2_ atmosphere. Agar plates were inspected at 24 h and a second time after 48 h of incubation. If growth was weak at 24 h, the incubation time was extended until sufficient colony material was available for further testing. Gps-like isolates (small, dewdrop-shaped growth on chocolate agar) were biochemically characterized by catalase, urease and CAMP phenomenon. If demanded by the customer, colonies were preserved at −80 °C in cryobanks (Cryobank, Mast Group Ltd., Bootle, United Kingdom) and since 2018 a PCR based on capsule genes has been performed for serotyping the strains [2]. 

### 4.2. Molecular Biological Detection of Pathogens by PCR, Sero- and Pathotyping of Gps

Direct pathogen detection from lung tissue (2 × 25 mg) was performed by extraction using the commercially available DNA (DNeasy Blood and Tissue Kit, Quiagen GmbH, Hilden, Germany) or RNA (RNeasy Blood and Tissue Kit, Quiagen GmbH, Hilden, Germany) extraction kits. After extraction, a pathogen-specific method determined the used protocol for mastermix and amplification.

Genome fragments of *M. hyopneumoniae* were visualized using a multiplex real-time PCR [51], while for detection of genome fragments of *M. hyorhinis*, a commercially available real-time PCR (BactoReal Kit Mycoplasma hyorhinis, Ingenetix GmbH, Vienna, Austria) was used [52]. 

PCV2-specific DNA fragments were detected with a TaqMan-based real-time PCR according to [53]. Detection of influenza A virus and PRRSV-1 (EU) and -2 (US) was based on commercially available PCR diagnostic kits. A differentiation of PRRSV-1 and -2 was possible with the used PRRSV RT-PCR (EZ-PRRSVTM MPX 4.0 assay, Tetracore R, Rockville, MD, USA) [54]. All known swine influenza subtypes were detected with a commercially available RT-PCR (EZ-Universal Flu A 2.0 RT-PCR, Tetracore R, Rockville, MD, USA) [55].

The amplification was carried out on a real-time cycler (Applied Biosystems 7500 Real Time PCR System, Thermo Fisher Scientific, Waltham, MA, USA). 

To prepare the samples, the bacterial cultures were first extracted manually using the lysis buffer Tris-HCL ph 8.5. Gps identification of isolates with PCR as well as serotyping were based on the primers described by [2]. The serotyping PCR included two steps with a first panel of primers targeting serovars 2, 3, 6, 7, 9, 10 and 11, and a second panel of primers targeting serovars 1, 4, 5, 8, 12, 13, 14 and 15 with each primer in a concentration between 60 and 400 nM in 25 μL. The respective master mix is composed as follows: 12.5 μL Qiagen Multiplex PCR Reaction Mix (Qiagen, Hilden, Germany), 3.0 μL Q Solution, 1.2 μL Primer Mix A or 1.55 μL Primer Mix B and 5.8 μL H2O (Primer-A) or 5.45 μL H20 (Primer-B). To each of these, a 2.5 μL DNA template is added. The cycling conditions for in total 30 cycles were as follows: 10 min at 94 °C, 30 s at 94 °C, 90 s at 59 °C, 90 s at 72 °C, 5 min at 72 °C. 

A subset of isolates, not only from 2018–2021 but also from earlier years and serotyped in other laboratories, was analysed by PCR to differentiate potential virulent Gps strains. This PCR was established according to published and modified methods of [2,6,7]. Primers AV1-F, V1-R and NV1-R were used with a final total concentration of 400 nM each. In addition, the Gps specific primers HPS_219690793-F and HPS_219690794-R were also added at a final concentration of 400 nM. The master mix per sample consists of 12.5 μL Quiagen PCR Reaction Mix, 5 μL Primer Mix, 5 μL H_2_O. This is added together with 2.5 μL of the DNA template. The amplification protocol with a total of 30 cycles was composed, respectively, as follows: 15 min at 95 °C, 45 s at 94 °C, 45 s at 57 °C, 60 s at 72 °C, 7 min at 72 °C. Subsequently, gel electrophoresis was performed in 4% agarose gel for 92 min at 170 volts. The Thermo Scientific GeneRuler 100 bp DNA Ladder was used as the DNA marker.

### 4.3. Antimicrobial Susceptibility Testing

As there is currently no standard sensitivity testing method for Gps, the determination of the MIC of the different antimicrobial substances followed available CLSI guidelines for other *Pasteurellaceae* and other pathogens. 

To determine the sensitivity of Gps to 14 antimicrobial agents, the most recent CLSI manuals for routine diagnostic procedures [56,57,58,59,60] were used. The optical density of colony material in a 5 mL 154 mM NaCl suspension was measured using a densitometer (bioMérieux Marcy l’Etoile, Marcy l’Etoile, France) and adjusted to McFarland 0.5 (corresponding to 10^6^–10^8^ CFU/mL). Ten milliliters of sterile Haemophilus test medium broth (HTM broth Thermo Scientific Sensitive™, Thermo Fisher Scientific, Waltham, MA, USA) was then mixed with 50 μL of the bacterial suspension and 50 μL of this suspension was pipetted into the wells of a commercial microtitre plate (Sensititre^®^ NLV 39, TREK Diagnostic Systems Ltd., Cleveland, OH, USA). This was then incubated at 35 ± 2 °C, 5% CO_2_ for 20–24 h.

Clinical breakpoints from the Clinical and Laboratory Standards Institute were used in the assessment of growth inhibition [58,59]. Other published clinical breakpoints in routine assessment procedures were used if breakpoints were not specifically available for Gps [61]. Thus, a clear classification into “resistant” (r), “intermediate” (i) or “sensitive” (s) was made (Table 9).

Wells of the microtiter plate are coated with antimicrobial substances in a two-fold dilution series as provided by the manufacturer and suggested by the German veterinary society working group on antimicrobial resistance [60]: ampicillin (0.12–32 mg/L), ceftiofur (0.12–8 mg/L), cephalothin (1–16 mg/L), colistin (0.5–4 mg/L), (enrofloxacin (0.03–2 mg/L), erythromycin (0.12–16 mg/L), florfenicol (1–8 mg/L), gentamicin (0.25–16 mg/L), penicillin G (0.06–16 mg/L), spectinomycin (4–64 mg/L), tetracycline (0.12–8 mg/L), tiamulin (8–32 mg/L), tilmicosin (1–32 mg/L), tulathromycin (2–64 mg/L). For each Gps isolate, the minimal inhibitory concentration (MIC) of the different antimicrobials was determined. Growth inhibition was interpreted and isolates were classified as “resistant” (r), “intermediate” (i) or “susceptible” (s) following CLSI-approved clinical breakpoints [38,58,59,61] or other published clinical breakpoints from routine evaluation procedures. For gentamycin and spectinomycin, the clinical breakpoint for *Pasteurella multocida* in cattle was used [62]. Clinical breakpoints for colistin were adopted from former DIN/AVID data [60]. 

### 4.4. Data Management and Statistical (Evaluation)

Entire laboratory test results were recorded in the laboratory information system LabControl© 2002, Ticono-Software, Hanover, promptly after a check according to the quality assurance system of the accredited laboratory at the Field Station for Epidemiology. For statistical data evaluation with SAS, version 9.4 (SAS Institute, Cary, NC, USA), the corresponding data set was exported to Excel, version 2016 (Microsoft Corporation, Albuquerque, NM, USA). If several Gps isolates were isolated from a farm within one year, only the first isolate was included in the data set. In the case of more than one Gps isolate from one farm in the entire period, these were considered to be independent of each other if they were isolated approximately one year apart. Relative frequencies of susceptible, intermediate and resistant isolates were deduced from MIC distributions of each antimicrobial substance. The MIC_50_- (median) and MIC_90_-(90%-percentile) values were calculated. For statistical evaluations, all isolates classified as intermediate or resistant to a respective antimicrobial according to the clinical cut-off were considered as resistant.

The time period 2006–2013 was regarded as the reference period for comparison with time period 2014–2021 to evaluate the time effect on the frequency of resistant Gps isolates.

Some pilot studies for monitoring AMU started already in 2011 and AMU in swine production started to decrease in 2013/2014. We therefore hypothesized that the reduction in AMU in swine production might be paralleled or followed by a decrease in frequencies of resistant Gps isolates. Frequencies of resistant isolates in the two eight-year time periods from 2006–2013 and 2014–2021 were therefore compared in uni- and multivariable logistic regression models. In a first step, the influence of the sampled age group, the organ-sampling site and the time period on the proportion of resistant isolates was analyzed by univariable logistic regression model analysis. The low numbers of Gps isolates originating from the nose and from gilts were excluded from statistical evaluation. The model was not appropriate for cephalothin and tetracyclin because for both substances all isolates were resistant in the time period 2006–2013. 

In a second step the variables age group of sampled pigs and sampling site were included into the model and a multivariable logistic regression model was finally performed to identify any impact of age group and sampling site on the temporal changes in the frequencies of resistant isolates simultaneously. The significance level for all statistical models was set at 0.05. No adjustment was made for multiple testing.

The frequencies of Gps serotypes and coinfecting agents in total and also in the different sampling sites were described.

## Figures and Tables

**Table 1:** Serotypes of Gps in a subset of isolates (*n* = 101) from the years 2018–2021.

Gps Serotype	Number of Isolates	Proportion of Isolates (%)
1	8	7.9
2	1	1.0
1/2	2	2.0
4	30	29.7
5/12	13	12.9
7	13	12.9
9	7	6.9
10	1	1.0
11	1	1.0
13	22	21.8
14	1	1.2
15	2	2.0

**Table 2:** Distribution of Gps isolates originating from different sampling sites and age groups (*n* = 2046 pigs).

Sampling Site	Suckling Piglets		Nursery Pigs		Fatteners		Sows		No Age Group		All Samples	
	*n*	%	*n*	%	*n*	%	*n*	%	*n*	%	*n*	%
Respiratory tract	118	73.3	1252	83.7	60	76.9	2	40.0	265	86.6	1697	82.9
Pericardium	18	11.2	104	7.0	5	6.4	2	40.0	10	3.3	139	6.8
Pleural cavity	8	5.0	59	3.9	4	5.1	−	−	13	4.2	84	4.1
Brain	7	4.3	44	2.9	7	9.0	−	−	12	3.9	70	3.4
Joint	6	3.7	18	1.2	2	2.6	1	20.0	1	0.3	28	1.4
Abdominal cavity	3	1.9	19	1.3	−	−	−	−	1	0.3	23	1.1
Nose	1	0.6	−	−	−	−	−	−	4	1.3		
Total	161	100	1496	100	78	100	5	100	306	100	2046	100

− No isolates avaliable.

**Table 3:** Distribution of a subgroup of *n* = 54 Gps strains tested for the *vta* gene.

Gps Isolates Positive for LS vta	Serotypes										
	1	4	5/12	7	9	11	13	14	15	n.s. *	Total
Respiratory tract	1	16	7	6	1	1	6	1	1	5	45
Other sites	−	−	2	2	1	−	2	−	−	2	9
Total	1 (1.9%)	16 (29.6%)	9 (16.7%)	8 (14.8%)	2 (3.7%)	1 (1.9%)	8 (14.8%)	1 (1.9%)	1 (1.9%)	7 (13.0%)	54 (100%)

All strains were found to be positive for the *vta* gene. * not serotyped. − not tested.

**Table 4:** Results of antimicrobial susceptibility testing of Gps isolates (*n* = 2046).

Substance (Clinical Breakpoint (mg/L))		Number of Isolates with MIC Values (mg/L) of												MIC_50_	MIC_90_	s	i	r
	*n*	≤0.03	0.06	0.12	0.25	0.5	1	2	4	8	16	32	≥64	(mg/L)		%	%	%
Ampicillin (≤0.5)	2046	−	−	1372	260	128	57	48	29	28	39	33	52	≤0.13	2.00	86.02	2.79	11.19
Ceftiofur (≤2)	2046	−	−	313	5	3	1670	13	6	−	36	−	−	1.00	1.00	97.95	0.29	1.80
Cephalothin ^1^ (≤8)	2046	−	−	−	−	−	291	16	1637	46	42	6	8	4.00	4.00	15.00	80.00	5.00
Colistin (<0.5)	2046	−	−	−	−	1868	91	16	4	67	−	−	−	0.50	0.50	91.30	5.20	3.50
Enrofloxacin (≤0.25)	2046	281	1503	108	68	63	12	8	3	−	−	−	−	0.06	0.13	95.80	3.10	1.10
Erythromycin (≤0.25)	2046	−	−	558	221	382	412	249	94	78	52	−	−	0.50	4.00	38.10	18.70	43.30
Florfenicol (≤2)	2046	−	−	−	−	−	1956	63	23	3	1	−	−	1.00	1.00	98.70	1.10	0.20
Gentamicin (≤2)	2046	−	−	−	66	28	79	1384	337	32	24	96	−	2.00	4.00	76.10	16.50	7.40
Penicillin G ^2^ (≤0.25)	2046	−	969	214	370	214	62	39	23	25	124	6	−	0.13	2.00	75.90	10.50	13.60
Spectinomycin ^3^ (≤32)	2046	−	−	−	−	−	−	−	277	1562	101	47	59	8.00	16.00	97.10	0.30	2.50
Tiamulin (≤16)	2046	−	−	−	−	−	−	−	1518	422	51	20	35	4.00	8.00	97.30	−	2.70
Tilmicosin (≤16)	2046	−	−	−	−	−	230	38	1557	73	65	30	53	4.00	8.00	95.94	−	4.10
Tetracycline (≤0.5)	2046	−	−	125	64	391	1121	100	66	48	65	65	−	1.00	4.00	28.40	54.80	16.80
Tulathromycin ^4^ (≤16)	325	−	−	−	−	−	−	272	25	10	11	5	2	2.00	4.00	97.80	1.50	0.60

Susceptibility and resistance of Gps isolates were assessed with respect to clinical breakpoints of the CLSI standards for Gps in swine. Bacterial isolates were categorized as “susceptible” (s), “intermediate” (i) or “resistant” (r). If no CLSI-approved clinical breakpoints for Gps were available, other published clinical breakpoints were used for assessment: ^1^ CLSI M100-S28 human, ^2^ CLSI VET08-A4 *Pasteurella multocida* swine, ^3^ CLSI VET08-A4 *Pasteurella multocida* cattle. ^4^ Tulathromycin was tested since 2017. The white area contains the dilution ranges tested. When isolates grew in the highest concentration of an antimicrobial agent, the corresponding MICs are considered to be equal to or higher than the next (not tested) concentration. − No isolates available.

**Table 5:** Univariable logistic regression model for comparison of resistant Gps isolates with respect to the sampling time period.

Risk Categories	Resistant		Susceptible		Univariable Model			
	*n*	%	*n*	%	OR	95% CI		*p*
						Lower	Upper	
Ampicillin								
2006–2013 (ref)	180	14.48	1063	85.52	1	x	x	x
2014–2021	106	13.20	697	86.80	0.898	0.694	1.163	0.4148
Ceftiofur								
2006–2013 (ref)	38	53.06	1250	96.94	1	x	x	x
2014–2021	4	0.50	799	99.50	0.159	0.056	0.447	0.0004
Cephalothin *								
2006–2013 (ref)	1243	100.00	0	0.00	1	x	x	x
2014–2021	496	61.77	307	38.23	n.d.	n.d.	n.d.	*
Colistin								
2006–2013 (ref)	105	8.45	1138	91.55	1	x	x	x
2014–2021	73	9.09	730	90.91	1.084	0.793	1.482	0.6141
Enrofloxacin								
2006–2013 (ref)	64	5.15	1179	94.85	1	x	x	x
2014–2021	22	2.74	781	97.26	0.519	0.317	0.849	0.009
Erythromycin								
2006–2013 (ref)	866	69.67	377	30.33	1	x	x	x
2014–2021	401	49.94	402	50.06	0.434	0.361	0.522	<0.0001
Florfenicol								
2006–2013 (ref)	21	1.69	1722	98.31	1	x	x	x
2014–2021	6	0.75	797	99.25	0.438	0.176	1.090	0.0760
Gentamicin								
2006–2013 (ref)	305	24.54	938	75.46	1	x	x	x
2014–2021	184	22.91	619	77.09	0.914	0.742	1.127	0.4006
Penicillin								
2006–2013 (ref)	304	24.46	939	75.54	1	x	x	x
2014–2021	189	23.54	614	76.46	0.951	0.772	1.171	0.6356
Spectinomycin								
2006–2013 (ref)	49	3.94	1194	96.06	1	x	x	x
2014–2021	10	1.25	793	98.75	0.307	0.155	0.610	0.0007
Tetracyclin *								
2006–2013 (ref)	1243	100.00	0	0.00	1	x	x	x
2014–2021	222	27.68	580	72.32	n.d.	n.d.	n.d.	*
Tiamulin								
2006–2013 (ref)	48	3.86	1195	96.14	1	x	x	x
2014–2021	7	0.87	796	99.13	0.219	0.099	0.486	0.0002
Tilmicosin								
2006–2013 (ref)	61	4.91	1182	95.09	1	x	x	x
2014–2021	22	2.74	781	97.26	0.546	0.332	0.896	0.0167

Univariable logistic regression analysis of proportion of resistant isolates to different antimicrobial substances with fixed effect time period (reference category is time period 2006–2013) as indicated by “ref.”. For tulathromycin, no data from the time period 2006–2013 existed. OR: point estimate/odds ratio, *p*: *p*-value of the Wald test, *n*: absolute number of isolates, %: percentage of isolates, n.d.: not determinable because model does not fit for this data set, * due to 100% resistant isolates in the time period 2006–2013 the statistical model cannot be applied to cephalothin and tetracyclin.

**Table 6:** Multifactorial logistic regression analysis with fixed effect time period and factors “age group” and “sampling site” with respect to colistin, erythromycin, florfenicol and tetracyclin.

	Colistin	**Resistant Isolates**		**Susceptible Isolates**		**Univariable log. reg.**		**Multivariable log. reg.**	
		*n*	%	*n*	%	OR	*p*	OR	*p*
**Time period**	**2006–2013** (Ref.)	105	8.45	1138	91.55	1	−	1	−
	2014–2021	73	9.09	730	90.91	1.08	0.614	1.00	0.989
Age group	**Nursery pigs (Ref.)**	124	8.29	1372	91.71	1	−	1	−
	Suckling piglet	13	8.07	148	91.93	0.97	0.925	0.91	0.755
	Fattening Pigs	6	7.69	72	92.31	0.92	0.852	0.87	0.759
	not specified	35	11.44	271	88.56	1.43	0.078	1.46	0.072
Sampling site	**Respiratory tract (Ref.)**	131	7.72	1566	92.28	1	−	1	−
	Pericardium	22	15.83	117	84.17	2.25	0.001	2.38	0.001
	Pleural cavity	11	13.10	73	86.90	1.80	0.080	1.81	0.079
	Brain	9	12.86	61	87.14	1.76	0.124	1.77	0.124
	Joints	3	10.71	25	89.29	1.43	0.559	1.60	0.450
	Abdominal cavity	1	4.35	22	95.65	0.54	0.552	0.57	0.585
	Erythromycin	**Resistant Isolates**		**Susceptible Isolates**		**Univariable log. reg.**		**Multivariable log. reg.**	
		*n*	%	*n*	%	OR	*p*	OR	*p*
Time period	**2006–2013 (Ref.)**	866	69.67	377	30.33	1	−	1	−
	2014–2021	401	49.94	402	50.06	0.43	<0.001	0.45	<0.001
Age group	**Nursery pigs (Ref.)**	917	61.30	579	38.70	1	−	1	−
	Suckling piglet	103	63.98	58	36.02	1.12	0.507	1.12	0.510
	Fattening Pigs	51	65.38	27	34.62	1.19	0.470	1.26	0.355
	not specified	193	63.07	113	36.93	1.08	0.561	0.946	0.682
Sampling site	**Respiratory tract (Ref.)**	1073	63.23	624	36.77	1	−	1	−
	Pericardium	75	53.96	64	46.04	0.68	0.031	0.79	0.201
	Pleural cavity	48	57.14	36	42.86	0.78	0.261	0.87	0.545
	Brain	39	55.71	31	44.29	0.73	0.204	0.80	0.385
	Joints	12	42.86	16	57.14	0.44	0.031	0.42	0.033
	Abdominal cavity	17	73.91	6	26.09	1.65	0.296	1.47	0.431
	Florfenicol	**Resistant Isolates**		**Susceptible Isolates**		**Univariable log. reg.**		**Multivariable log. reg.**	
		*n*	%	*n*	%	OR	*p*	OR	*p*
Time period	**2006–2013 (Ref.)**	21	1.69	1222	98.31	1	−	1	−
	2014–2021	6	0.75	797	99.25	0.44	0.076	0.42	0.062
Age group	Nursery pigs (Ref.)	19	1.27	1477	98.73	1	−	1	−
	Suckling piglet	1	0.62	160	99.38	0.49	0.483	0.48	0.471
	Fattening Pigs	4	5.13	74	94.87	4.20	0.011	4.29	0.009
	Not specified	3	0.98	303	99.02	0.77	0.675	0.696	0.564
Sampling site	Respiratory tract (Ref.)	25	1.47	1672	98.53	1	−	1	−
	Pericardium	−	−	139	100.00	−	−	−-	−
	Pleural cavity	2	2.38	82	97.62	1.63	0.510	−	−
	Brain	−	−	70	100.00	−	−	−	−
	Joints	−	−	28	100.00	−	−	−	−
	Abdominal cavity	−	−	23	100.00	−	−	−	−
	Tetracyclin	**Resistant Isolates**		**Susceptible Isolates**		**Univariable log. reg.**		**Multivariable log. reg.**	
		*n*	%	*n*	%	OR	*p*	OR	*p*
Time period	2006–2013 (Ref.)	1243	100.00	−	−	−	−	−	−
	2014–2021	222	27.68	580	72.32	1	−	1	−
Age group	Nursery pigs (Ref.)	1044	69.83	451	30.17	1	−	1	−
	Suckling piglet	120	74.53	41	25.47	1.26	0.216	1.32	0.146
	Fattening Pigs	56	71.79	22	28.21	1.10	0.713	1.16	0.560
	Not specified	240	78.43	66	21.57	1.57	0.003	1.66	0.001
Sampling site	Respiratory tract (Ref.)	1248	73.58	448	26.42	1	−	1	−
	Pericardium	81	58.27	58	41.73	0.50	<0.001	0.50	0.0001
	Pleural cavity	55	65.48	29	34.52	0.68	0.103	0.68	0.097
	Brain	41	58.57	29	41.43	0.51	0.006	0.49	0.005
	Joints	20	71.43	8	28.57	0.90	0.798	0.86	0.725
	Abdominal cavity	19	82.61	4	17.39	1.71	0.334	1.78	0.297

Reference categories for the logistic regression method (time period 2006–2013, nursery pigs, respiratory tract) are highlighted in bold and indicated by “Ref.”. Univariable log. reg.: One-factorial logistic regression model, multivariable log.reg.: Multi-factorial logistic regression model, OR: point estimate/odds ratio, *p*: *p*-value of the Wald test, *n*: absolute number of isolates, %: proportion of isolates. −No isolates available.

**Table 7:** Proportion of resistant Gps isolates from the respiratory tract of nursery pigs in the time periods 2006–2013 and 2014–2021.

Antimicrobial Substance	2006–2013				2014–2021				*p* *
	Resistant Isolates		Sensitive Isolates		Resistant Isolates		Sensitive Isolates		
	Number	(%)	Number	(%)	Number	(%)	Number	(%)	
Ampicillin	105	13.74	659	86.26	64	13.11	424	86.89	0.751
Ceftiofur	24	3.14	740	96–86	4	0.82	484	99.18	0.012
Cephalothin	764	100.00	−	−	288	59.02	200	40.98	n.d.
Colistin	50	6.54	714	93.46	40	8.20	448	91.80	0.271
Enrofloxacin	33	4.32	731	95.68	15	3.07	473	69.93	0.265
Erythromycin	540	70.68	224	29.32	244	50.00	244	50.00	<0.0001
Florfenicol	13	1.70	751	98.30	5	1.02	483	98.98	0.331
Gentamicin	186	24.35	578	75.65	119	24.39	369	75.61	0.987
Penicillin	173	22.64	591	77.36	112	22.95	376	77.05	0.899
Spectinomycin	28	3.66	736	96.34	6	1.23	482	98.77	0.014
Tetracyclin	764	100.00	−	−	133	27.31	354	72.69	n.d.
Tiamulin	33	4.32	731	95.68	4	0,82	484	99.18	0.001
Tilmicosin	38	4.97	726	95.03	15	3.07	473	96.93	0.107

* Significant differences according to linear logistic regression models. − No isolates available.

**Table 8:** Microorganisms in samples positive for Gps.

Additional Microorganisms in Samples Positive for Gps		Combinations with Gps				Number of Samples Tested
		Negative		Positive		
		*n*	%	*n*	%	*n*
Type	Pathogen					
Virus ^a^	Influenza	730	77.00	218	23.00	948
	PCV2	633	82.00	139	18.00	772
	PRRSV-EU	818	74.9	274	25.10	1092
	PRSV-US	977	90.70	100	9.30	1077
Bacteria	Actinobacillus pleuropneumoniae *	2000	97.80	46	2.20	2046
	Actinobacillus suis *	2045	100.00	1	0.00	2046
	Beta-haemolytic streptococcus *	1992	97.40	54	2.60	2046
	Bordetella bronchiseptica *	1824	89.10	222	10.90	2046
	*Mycoplasma hyopneumoniae ^a^*	739	86.80	112	13.20	851
	*Mycoplasma hyorhinis ^a^*	308	54.90	253	45.10	561
	Staphylococcus aureus *	2016	98.50	30	1.50	2046
	Streptococcus suis *	1526	74.60	520	25.40	2046
	Pasteurella multocida *	1873	91.20	173	8.50	2046

^a^ detection by PCR, * detection by cultural microbiological methods.

**Table 9:** The used clinical cut-offs for *Glaesserella parasuis*.

Antimicrobial Substance	Sensitive	Intermediate	Resistant
Ampicillin	≤0.5	1	≥ 2
Ceftiofur	≤2	4	≥8
Cephalotin	≤2	4	≥8
Colistin	≤0.5	1–2	≥4
Enrofloxacin	≤0.25	0.5	≥1
Erythromycin	≤0.25	0.5	≥1
Florfenicol	≤2	4	≥8
Gentamicin	≤2	4	≥8
Penicillin	≤0.25	0.5	≥1
Spectinomycin	≤32	64	>64
Tetracyclin	≤0.5	1	≥2
Tiamulin	≤16	−	≥32
Tilmicosin	≤16	−	≥32
Tulathromycin	≤16	32	≥64

− not specified.

## Data Availability

The datasets used and/or analysed during the current study are available from the corresponding author on reasonable request.
